# Feasibility and accuracy of robot-assisted tunnel placement in anatomic arthroscopic posterior cruciate ligament reconstruction

**DOI:** 10.1097/JS9.0000000000001936

**Published:** 2024-07-11

**Authors:** Gang Yang, Hong-Jie Huang, Jia-Yi Shao, Ding-Ge Liu, Kai-Ping Liu, Zhi-Hua Zhang, Lang-Ran Wang, Qi-Ning Wang, Zhi-Hao Zhou, Jian-Quan Wang, Xin Zhang

**Affiliations:** a Department of Sports Medicine, Peking University Third Hospital, Institute of Sports Medicine of Peking University; b Beijing Key Laboratory of Sports Injuries; c Engineering Research Center of Sports Trauma Treatment Technology and Devices, Ministry of Education; d Department of Advanced Manufacturing and Robotics, Peking University; e Institute for Artificial Intelligence, Peking University, Beijing, People’s Republic of China

Highlights

- Robotic surgery created bone tunnels with superior anatomical precision compared to conventional surgery.
- Robot-assisted drilling facilitated more accurate drilling of the bone tunnel according to the plan.
- Robot-assisted surgery may yield promising results for posterior cruciate ligament reconstruction.

*Dear Editor,*

The posterior cruciate ligament (PCL) plays a crucial role in maintaining the kinematics and stability of the knee joint^1^. The accurate placement of the tibial and femoral tunnels is a key step in anatomical PCL reconstruction^2^. Nonanatomical placement of the tibial tunnel leads to greater posterior tibial translation^3^. Furthermore, the popliteal artery may be injured if the placement of the tibial tunnel is deep, as the tibial insertion of the PCL is close to that of the popliteal artery, thereby resulting in serious complications^4,5^. Handheld drill guides are commonly used to locate and drill tunnels under arthroscopic guidance during PCL reconstruction. However, creating an accurate bone tunnel using only an arthroscopic system is difficult, even for experienced surgeons. Therefore, this study aimed to use robot-assisted PCL reconstruction of bone tunnels and compare it with that of traditional surgical methods. It was hypothesized that the use of the surgical robot system would yield a more accurate placement of the bone tunnel and more anatomic reconstructions.

This study was approved by the Medical Science Research Ethics Committee of our hospital (approval number: M2019056). The specimens used in this study were donated by the anatomy program of our university. And consecutive patients who had undergone PCL reconstruction performed by the same senior author at our center between January 2022 and November 2023 were included in this study to compare the accuracy of traditional surgery. The orthopedic surgical robot (TiRobot, TINAVI Medical Technologies Co., Ltd.) with a specific cruciate ligament software system was used in this study. TiRobot comprises a 6-degrees of freedom (DOF) robot arm (with a guide sheath at the end to pass Kirschner wires), an optical tracking device to track the position of the robotic arm and the knee joint, a surgical planning and controlling workstation, and special infrared trackers to perform the positioning function (Fig. 1A).

**Figure 1:**
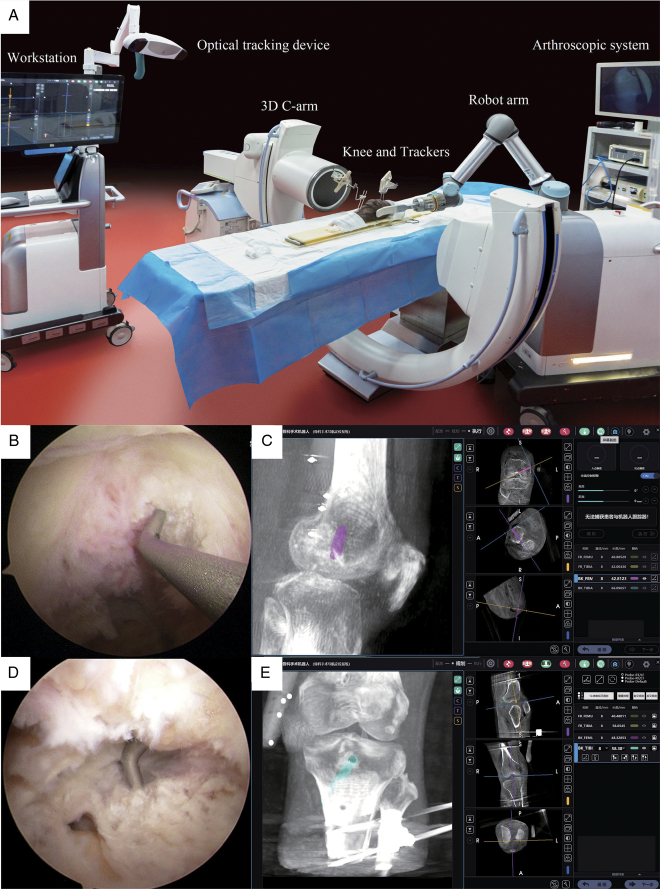
The composition of the robot and intraoperative planning process (a right knee was taken as an example). (A) The composition of the orthopedic surgical robot system and the placement of all the major equipment required for surgery. (B) The locator was placed into a knee via the anteromedial portal and was directed to the femoral insertion site of the posterior cruciate ligament (PCL). (C) The planned location of the PCL femoral tunnel was determined on the workstation. (D) The tibial insertion site of the PCL was marked by the locater via the postmedial portal. (E) The PCL tibial tunnel was determined on the workstation.

The robotic surgical approach was as follows: the knee joint was scanned with a Three-dimensional (3D) C-arm (Cios Spin, Siemens), and the 3D images of the knee joint were then transmitted to the surgical planning and control workstation of the robot system to complete the image registration for preoperative planning. The knee joint was flexed at 90°, a locator with an infrared tracker was used to determine the location of the PCL tibial footprint and the anterolateral bundle of the femoral footprint. These points were subsequently registered in the system as the exit points for the tunnel. The entrance point to the tunnel was selected on the skin in contact with the locator and registered with the system. The system automatically registered the position of the planned tunnel following the registration of the entry and exit points (Fig. 1B to E). The location and length of the planned tunnels were displayed on the workstation and recorded by an assistant after the adjustment. The foot pedal was pressed by the surgeon to activate the 6-DOF robot arm, which automatically adjusts the position at the end of the robotic arm. The PCL tunnels were drilled along the guide sheath using a 2 mm Kirschner wire, followed by an 8 mm femoral drill, after adjusting the guide sheath. Arthroscopy facilitated access to the joint cavity at any time during and after tunnel drilling was completed to evaluate the damage to the intra-articular structures (Fig. 2A and D).

**Figure 2:**
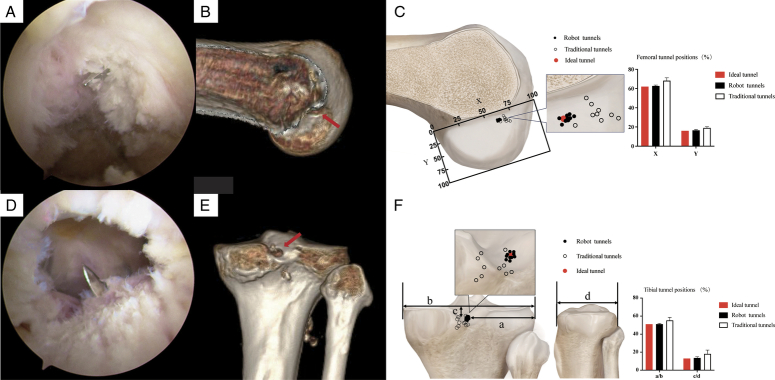
The drilling process (a right knee was taken as an example). (A) The Kirschner wire reached the planned position of the femur, which is the femoral insertion point of the PCL. (B) The location of the femoral tunnel (the point marked by the red arrow) was confirmed by 3D computed tomography (CT). (C) Measurement of the femoral tunnel position. A rectangular coordinate system was established below the Blumensaat line. In this rectangle, *x* is located at the Blumensaat line, and *y* is perpendicular to *x* and tangent to the posterior margin of the medial femoral condyle. The scatter points represent the locations of the femoral tunnels. The bar graph represents the mean value of the femoral tunnel position. (D) The Kirschner wire reached the planned position of the tibia, which is the tibial insertion point of the PCL. (E) The location of the tibial tunnel (the point marked by the red arrow) was confirmed by 3D CT. (F) Measurement of the tibial tunnel position. The anteroposterior and mediolateral diameters of the tibial plateau were used to indicate the location of the tibial tunnel: a represents the distance between the tibial tunnel and the lateral edge of the tibial plateau, b represents the mediolateral diameter of the tibial plateau, c is the distance between the tibial tunnel and the horizontal tangent line of the medial tibial plateau, and d is the anteroposterior diameter of the tibial plateau. The scatter points represent the locations of the tibial tunnels. The bar graph represents the mean value of the tibial tunnel position.

Traditional PCL reconstructions were performed by the same surgeon in all patients, the surgical technique was performed as described in a previous study^6^.

3D computed tomography (CT) was performed postoperatively to examine the knee joints. The location and length of the PCL tunnel were recorded (Fig. 2B and E). The location of the PCL and measurement methods refer to previous research^7,8^. Ten specimens were considered sufficient based on the findings of previous studies^9,10^. The normal distribution of the variables was confirmed using the Shapiro–Wilk test. Normally distributed data were compared using a *t*-test; the Wilcoxon signed-rank test was used otherwise. A *P-*value of <0.05 was considered statistically significant.

A total of 10 freshly frozen human knee specimens and 10 patients were measured in this study (Table 1). No significant difference was observed between the position of the tunnel established by the robot and the anatomical position of the insertion of PCL (*P*>0.05) or the position planned during the surgery (*P*>0.05). However, the position of the femoral tunnel established via traditional surgery differed significantly from the anatomical position (*P*<0.05). Therefore, the position of the tunnels established via robotic surgery were more anatomical and less variable (Fig. 2C and F). In the robotic surgery, there was no significant difference between the actual and planned bone tunnel lengths (*P*>0.05), and the maximum error between the two did not exceed 2 mm.

**Table 1:** Baseline demographic and comparison of robotic and traditional surgery.

Characteristics	Robot surgery		Traditional surgery	
Laterality				
Left	4		5	
Right	6		5	
Sex				
Female	5		4	
Male	5		6	
Age (years)	60.9±5.2		39±12.5	
Operation time (min)	74.6±4.34		54.5±6.9	
	Tunnel position (%)			
Measurements	Anatomy	Robotic		Traditional tunnel
		Planned tunnel	Actual tunnel	
X	62‡	62.4±1.6	62.7±0.9†	68.2±3.0† ‡
Y	16‡	16.8±1.2	16.5±1.0	19.0±3.8‡
A/B	51‡	51.5±0.9	51.1±0.7†	55.3±3.2† ‡
C/D	13‡	13.1±1.3	13.6±1.3†	18.1±4.3† ‡
		Tunnel length (mm)		
Positions		Planned tunnel	Actual tunnel	Error
Femur		44.5±4.8	45.1±4.8	1.5±0.2 (1.2–1.9)
Tibia		55.3±4.8	54.4±5.0	1.2±0.3 (0.8–1.7)

X represents the femoral tunnel position along the Blumensaat line.

Y represents the position of the femoral tunnel perpendicular to the Blumensaat line.

A represents the distance between the exit center of the tibial tunnel and the lateral edge of the tibial plateau.

B represents the transverse diameter of the tibial plateau.

C represents the distance between the exit center of the tibial tunnel and the tangent line of the medial tibial plateau.

D represents the sagittal diameter of the tibial plateau.

† Significant difference between the position of the bone tunnel in traditional and robotic surgery (*P*<0.05).

‡ Significant difference between the position of the bone tunnel in traditional surgery and the anatomical insertion site position of the PCL (*P*<0.05).

This is the first cadaver study of robot-assisted PCL reconstruction. The main finding of this study was that the bone tunnel established via robotic surgery was more anatomical and more consistent than that established via traditional surgery. And robot-assisted surgery was effective in preventing popliteal artery injury. Because the surgeon can use the robotic system to plan the tibial tunnel length in advance. In addition, the radiation generated by the robot-assisted PCL reconstruction is acceptable, and the 3D C-arm that generated all ionizing radiation was used only once in each operation.

Our study had some limitations. First, cadaveric specimens were compared with those of clinical patients. Second, this study only demonstrated that robot-assisted PCL reconstruction is more accurate than traditional surgery and does not apply it in clinical practice. In the future, large sample-size studies on the effects of robotic surgery and traditional surgery on knee function will provide more sufficient and powerful evidence.

In conclusion, anatomical PCL reconstruction was performed using a surgical robot in the present study. The use of intraoperative planning based on 3D images and automatic navigation of the robotic arm resulted in a more anatomical and consistent placement of the bone tunnel. Robot-assisted surgery may yield promising results for PCL reconstruction.

## Ethical approval

This study was approved by the Peking University Third Hospital Medical Science Research Ethics Committee (M2019056).

## Consent

Written informed consent was obtained from the patient for publication of this case report and accompanying images. A copy of the written consent is available for review by the Editor-in-Chief of this journal on request.

## Source of funding

This research was supported by the National Natural Science Foundation of China (no. 82172447), the Beijing Science and Technology Program (no. Z221100007422020), the Key Research and Development Program of Shaanxi Province (no. 2023-YBSF-326), the Peking University Medicine Sailing Program for Young Scholars’ Scientific & Technological Innovation (BMU2023YFJHPY019), and the Beijing Natural Science Foundation (no. L202054).

## Author contribution

G.Y.: conceptualization, data curation, formal analysis, methodology, software, validation, writing – original draft, and writing – review and editing; H.-J.H.: conceptualization, methodology, software, and writing – review and editing; J.-Y.S.: data curation, formal analysis, methodology, software, and writing – review and editing; D.-G.L.: writing – review and editing; K.-P.L.: data curation, methodology, and writing – original draft; Z.-H.Z.: data curation; L.-R.W.: methodology; Q.-N.W.: software; Z.-H.Z.: software; J.-Q.W.: resources, supervision, and writing – review and editing; X.Z.: conceptualization, formal analysis, funding acquisition, investigation, methodology, project administration, resources, software, supervision, and writing – review and editing.

## Conflicts of interest disclosure

All authors have no financial conflicts of interest to disclose concerning this research.

## Research registration unique identifying number (UIN)

In this study, cadaveric studies were performed and previous surgical images were retrospectively analyzed; human experiments were not performed, so it was not applicable.

## Guarantor

Xin Zhang.

## Data availability statement

All datasets generated during and/or analyzed during the current study are available upon reasonable request.

## Provenance and peer review

None.

## Presentation

None.
